# Efficacy of levetiracetam in the treatment of pediatric epilepsy: A protocol for systematic review and meta-analysis

**DOI:** 10.1097/MD.0000000000028882

**Published:** 2022-02-25

**Authors:** Qiming Pang, Bangtao Li, Suli Zhang, Jiaoyang Li, Shuo Gu

**Affiliations:** Department of Neuroscience, Hainan Women and Children's Medical Center, Hainan, China.

**Keywords:** adverse effects, efficacy, levetiracetam, meta-analysis, pediatric epilepsy

## Abstract

**Background:**

To systematically collect, critically evaluate, and synthesize current evidence with respect to the efficacy, safety, and tolerability of levetiracetam as mono- or adjunctive therapy for children and adolescents with all types of epilepsy.

**Methods:**

The presentation of methods and results in this systematic review was performed according to the evaluation guidelines for health care interventions provided in the Preferred Reporting Items for Systematic Reviews and Meta-Analyses Protocol. Literature retrieval will use the Cochrane Library, Web of Science, PubMed, Embase, Allied and Complementary Medicine Database, China Biomedical Literature Database, China National Knowledge Infrastructure, China Science and Technology Journal Database, Wanfang Database, and Ongoing Clinical Trials Database.

The risk of bias of included studies is estimated by taking into consideration the characteristics including random sequence generation, allocation concealment, blinding of patients, blinding of outcome assessment, completeness of outcome data, selective reporting and other bias by Cochrane Collaboration's tool. Data synthesis and analyses are performed using RevMan 5.4 software.

**Results:**

The results of this systematic review and meta-analysis will be published in a peer-reviewed journal.

**Conclusion:**

Levetiracetam seems to be effective and safe for the treatment of pediatric epilepsy.

## 1 Introduction

Epilepsy is one of the most common medical problems that affect infants and children.^[1,2]^ An estimated 5% of children will have a seizure.^[3]^ A recent meta-analysis study found that the active period prevalence of epilepsy (the number of annual new and existing cases of epilepsy) was 4.8/1000 worldwide and in the USA was 0.94/1000 for children less than 18 years of age.^[4]^ Epilepsy and the conditions that cause epilepsy impact children and their families in many different ways, impacting cognition, behavior, and socioeconomic status.^[5,6]^ Uncontrolled epilepsy and the conditions that cause seizures leave indelible changes that can affect a child for life and even increase the risk of sudden death.

Currently, there is no ideal antiepileptic drug (AED) that can satisfy all the requirements of efficacy, safety, and tolerability, because almost all of the AEDs available can cause short- or long-term side effects or complication and some may be very serious. Levetiracetam (LEV), an antiepileptic drug, has become widely used in the treatment of several types of epilepsy.^[7,8]^ It is used as an adjunctive therapy in the treatment of partial onset, myoclonic, and/or primary generalized tonic-clonic seizures. In children, LEV is approved for use as adjunct treatment for partial epilepsy in patients aged 4 years and older, as well as for juvenile myoclonic epilepsy in patients aged 12 years and older.^[9]^

Although levetiracetam's exact mechanism of action is not clear, it seems that it differs from other AEDs on structure and function when binding to SV2A, which may play a role in the antiepileptic process.^[10]^ To gain more insight into this topic, we performed a protocol for systematic review and meta-analysis to evaluate clinical efficacy, safety, and tolerability of levetiracetam as mono- or adjunctive therapy in the treatment of children and adolescents with epilepsy.

## 2 Methods

### 2.1 Study registration

The protocol of this review was registered in OSF (OSF registration number: 10.17605/OSF.IO/V47KT). It is reported to follow the statement guidelines of preferred reporting items for systematic reviews and meta-analyses protocol.^[11]^ Since this study is on the basis of published studies, ethical approval is not required.

### 2.2 Searching strategy

This study will use the Cochrane Library, Web of Science, PubMed, Embase, Allied and Complementary Medicine Database, China Biomedical Literature Database, China National Knowledge Infrastructure, China Science and Technology Journal Database, Wanfang Database, and Ongoing Clinical Trials Database. There is no definite time limit for the retrieval literature, and the languages are limited to Chinese and English. We will consider articles published between database initiation and December 2021. The following Medical Subject Headings (MeSH) terms and free text were used: “levetiracetam,” “pediatric epilepsy,” and “randomized controlled trial”.

### 2.3 Inclusion and exclusion criteria

Selection criteria included in our meta-analysis were randomized controlled trials (RCTs), subjects confined to children and adolescents aged less than 16 years, diagnosis of epilepsy, treated with levetiracetam and compared to other AEDs or placebo, and provision of at least 2 outcomes of interest from seizure-free rate, seizure-frequency reduction from baseline (≥50%) and side effects. Exclusion criteria were studies comprising both children and adults that did not give results separately, case reports, retrospective studies, studies written in a language other than English or Chinese, and studies with samples less than 30.

### 2.4 Data selection

First, 2 investigators used Endnote X9 software to conduct a preliminary assessment of the title and abstract of each document in the database based on the established criteria for inclusion in the study to select eligible studies. After a preliminary assessment, the full text of the selected literature was evaluated, and the uncontrolled study, no randomization, inconsistent evaluation criteria, and similar data were excluded. Finally, the final included literature was exchanged and checked by researchers. If the 2 researchers disagree on the results of a study or eventual inclusion, we will resolve it through discussion or consultation with a third person.

### 2.5 Data extraction

Before data collection, the study team built a data extraction sheet. Two authors separately collected relevant information from each eligible study. The data extraction table mainly includes the following contents: research title, first author, year of publication, sample size, duration of disease, intervention measures, outcome indicators, adverse reactions, and so on. If a study has unclear or inadequate information, we will attempt to contact the authors via email.

### 2.6 Risk of bias assessment

Two investigators will separately assess the risk of bias of the included studies using the Cochrane risk of bias assessment tool. The evaluation of each study mainly included the following 7 aspects: random sequence generation, allocation hiding, blinding of participants and personnel, blinding of outcome assessment, incomplete outcome data, incomplete outcome data, selective outcome reporting, and other biases. Finally, the bias of the study will be rated on 3 levels: “low,” “high,” and “ambiguous”. These even domains will be separately appraised by 2 reviews, and discrepancies will be addressed by consulting a third reviewer.

### 2.7 Statistical analysis

In this study, we will apply RevMan 5.4 software for statistical analysis. The risk ratio and 95% confidence intervals were collected for enumeration data, while the mean difference or standardized mean difference and 95% confidence intervals were used to calculate continuous outcome data. The heterogeneity of the data was tested by calculating *I*^2^ statistics. The study was not considered to have a large heterogeneity when the *I*^2^ value was less than 50%. When the *I*^2^ value exceeded 50%, there was significant statistical heterogeneity among the trials. When there is homogeneity in the merged outcome results across sufficient studies, a meta-analysis will be conducted. Otherwise, we performed a subgroup analysis to explore the causes of the heterogeneity.

## 3 Discussion

Epilepsy is a common chronic disorder that requires long-term AED therapy.^[12,13]^ Approximately one half of patients fail the initial AED and about 35% are refractory to medical therapy, highlighting the continued need for more effective and better tolerated drugs.^[14]^ LEV has been demonstrated effective as adjunctive therapy for refractory partial-onset seizures, primary generalized tonic-clonic seizures, and myoclonic seizures.^[15,16]^ It is a unique AED that has multiple mechanism of action that differentiates it from conventional AEDs. But in recent years, apart from the most frequent adverse effects of LEV, such as nausea, gastrointestinal symptoms, dizziness, irritability and aggressive behavior, some rare adverse effects of LEV have been reported, including eosinophilic pneumonia, rhabdomyolysis, thrombocytopenia, elevated kinase and reduced sperm quality.^[17–19]^ However, evidence for a relationship between LEV serum concentrations and risk of adverse effects remains unclear. Further larger, prospective studies will be required to investigate the safety of LEV in the treatment of pediatric epilepsy

## Author contributions

**Conceptualization:** Bangtao Li, Jiaoyang Li.

**Formal analysis:** Suli Zhang.

**Funding acquisition:** Shuo Gu.

**Investigation:** Bangtao Li.

**Methodology:** Suli Zhang.

**Supervision:** Jiaoyang Li.

**Writing – original draft:** Qiming Pang.

**Writing – review & editing:** Shuo Gu.
